# Fitness-for-Purpose Assessment of Methods for Glyphosate Determination in Food: Trade-Off Between Analytical Performance and Environmental Impact

**DOI:** 10.3390/foods15030576

**Published:** 2026-02-05

**Authors:** Biancamaria Ciasca, Veronica Ghionna, Ivan Pecorelli, Emanuela Verdini, Antonio Moretti, Veronica Maria Teresa Lattanzio

**Affiliations:** 1 Institute of Sciences of Food Production, National Research Council of Italy, 70126 Bari, Italy; 2 Chemistry Department, Istituto Zooprofilattico Sperimentale dell’Umbria e delle Marche “Togo Rosati”, 06126 Perugia, Italy

**Keywords:** glyphosate, analytical method validation, environmental sustainability, pesticide analysis, screening method, quantitative method

## Abstract

Selecting analytical methods for pesticide residues in food increasingly requires balancing regulatory compliance, analytical performance, and environmental sustainability. This study presents a decision-support tool that evaluates the fitness-for-purpose of pesticide analytical methods by integrating SANTE/11312/2021 v2 validation criteria with Analytical GREEnness (AGREE)-based environmental metrics. Implemented in Excel with VBA macros, the tool guides users through the input of method parameters for both quantitative and screening approaches, scoring each against acceptance criteria. Based on the results, methods are classified as suitable for risk assessment, official control, or self-monitoring. The tool also calculates greenness scores to assess environmental impact. Glyphosate analysis in cereals was selected as a case study, and three approaches were compared: liquid chromatography coupled to tandem mass spectrometry (LC-MS/MS), flow-injection coupled to MS/MS (FI-MS/MS), and lateral flow assay (LFA). LC-MS/MS was identified as the only method suitable for official control, while FI-MS/MS and LFA met requirements for self-monitoring. The greenness assessment highlighted substantial differences, with LFA showing the lowest environmental footprint (AGREE scores of 0.63 and 0.68 for manual and automated LFAs). Overall, the tool provides a practical, user-friendly framework for selecting analytical methods that optimize both analytical performance and environmental sustainability, supporting informed decision-making in food testing.

## 1. Introduction

The selection of analytical methods for pesticide residues in food increasingly requires meeting not only analytical performance and regulatory fitness-for-purpose but also environmental sustainability in line with European Union (EU) initiatives such as the Green Deal [1] and the 2030 Agenda [2]. In this context, glyphosate analysis represents a timely and relevant case study, as its widespread use and strict regulatory requirements demand frequent monitoring through analytical approaches that differ markedly in complexity and environmental footprint, making it particularly suitable to evaluate the balance between fitness-for-purpose and environmental sustainability.

The regulation of glyphosate in the EU is currently governed by a 10-year approval period extending until 15 December 2033 [3,4,5]. This renewal was decided by the European Commission following comprehensive safety assessments conducted by the European Food Safety Authority (EFSA), the European Chemicals Agency (ECHA), and regulatory experts from EU Member States [4,5]. The approval includes restrictions such as prohibiting pre-harvest use as a desiccant but allows pre-harvest weed control. Member states must implement mitigation measures such as buffer strips to reduce drift risks [1,2]. The Commission can review glyphosate’s approval at any time if new evidence indicates it no longer meets safety criteria set by EU legislation [3,4,5].

Despite EFSA’s conclusions, non-governmental organizations have raised concerns about environmental and health impacts. Glyphosate can directly affect specific species and indirectly threaten ecosystems through impacts on biodiversity. Its dissipation and ecological impact are strongly influenced by soil microbial activity and site-specific soil conditions [6]. In addition, the development and engineering of crops that tolerate glyphosate’s herbicidal action, such as maize and soybeans, has increased its application, particularly in the United States, Brazil, Argentina, and Canada. Widespread glyphosate use has raised concerns about residues in food and potential dietary exposure, especially in vulnerable populations [7], driving demand for glyphosate-free certifications and products. For instance, the glyphosate-free certification market in North America increased by 170% in 2023 [8]. Several companies are progressively transitioning their product lines toward glyphosate-free offerings, thereby increasing the need for analytical methods capable of reliably supporting both regulatory compliance and voluntary certification.

The European regulatory framework for pesticides is among the strictest worldwide and has important implications for food safety controls [9]. Maximum residue levels (MRLs) for glyphosate in food of plant origin are currently set within the EU at 0.05–0.1 mg/kg [10], while the Codex Alimentarius Commission and the US Environmental Protection Agency established MRLs in the range of 0.05–40 mg/kg [11]. To ensure food safety and compliance, analytical methods must be tailored to specific operational purposes, which include (i) official control to enforce regulatory standards, (ii) self-monitoring by food companies to meet certification and consumer expectations, and (iii) risk assessment and ongoing monitoring to evaluate long-term exposure risks [12,13]. Analytical methods must therefore be selected according to their intended operational purpose, with performance requirements varying depending on the specific application. For pesticides, SANTE 11312/2021 v2 guidelines provide criteria for method validation and the monitoring of pesticide residues in food and feed [14].

A variety of analytical approaches are available for glyphosate determination, including liquid chromatography coupled with mass spectrometry (LC-MS/MS), ion chromatography coupled with mass spectrometry (IC-MS/MS), and immunoassay-based screening tests, such as enzyme-linked immunosorbent assay or immunochromatographic strip tests [15,16,17,18,19,20,21,22,23,24,25,26]. The latter are available on the market for self-monitoring purposes; however, to the best of our knowledge, validation data and information on their applicability in real operational conditions have not yet been reported.

A non-comprehensive overview of the main methods made available in the literature over the last 10 years is reported in Table 1. These methods differ in sensitivity, throughput, and suitability for specific applications, highlighting the need to select the appropriate method based on its intended operational purpose.

Increasing attention to the environmental sustainability of analytical procedures requires laboratories and regulatory bodies to balance analytical performance and regulatory compliance with environmental impact and practical operational constraints. While international guidelines support the development and validation of analytical methods for regulatory purposes, they do not fully address “green” aspects or their relevance across different operational contexts, leaving a gap in the simultaneous evaluation of method suitability and environmental impact. Existing tools provide partial solutions: metrics such as the Analytical Eco-Scale [27], GAPI [28], Analytical GREEnness (AGREE) [29], and Life Cycle Assessment focus only on environmental impact. White Analytical Chemistry (WAC) [30] allows a holistic comparison of analytical methods for different classes of food contaminants [31], integrating “green principles” with technical performance parameters (scope, limits of detection and quantification, precision, and accuracy) and practical aspects (cost, time efficiency, and simplicity) However, WAC does not explicitly evaluate method suitability with respect to the specific operational contexts.

To fill these gaps, the present study introduces a novel decision-support tool designed to be applicable to pesticide analytical methods, as it integrates SANTE validation criteria [14] with AGREE-based environmental metrics [29], allowing the simultaneous assessment of fitness-for-purpose and ecological performance. The tool helps users identify regulatory compliance, performance gaps, and opportunities for environmental improvement, providing guidance for adapting methods to different purposes.

The applicability of the tool was tested using glyphosate as a case study by assessing and comparing three analytical methods for glyphosate analysis in cereals: LC-MS/MS and flow-injection MS/MS (FI-MS/MS) for quantitative analysis, and a lateral flow assay (LFA) for screening purposes. The assessment highlighted the most critical factors requiring improvement, either to adapt a method for a different purpose or to enhance its ecological performance. The ecological impact of automation on LFA was also evaluated (automated LFA). The tool is provided as an open-access resource (Supplementary File S1: ToolKit Fit4Purpose&GreenEvaluation.).

## 2. Materials and Methods

### 2.1. Tool Description

**Figure 1:**
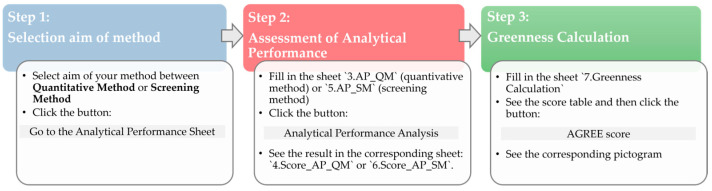
Workflow of the tool for analytical performance evaluation and greenness assessment. The process consists of three main steps, each corresponding to a differently colored Excel sheet. In Step 1, “Quantitative method” and “Screening method” are shown in bold as selectable options. The grey rectangle indicates the VBA macro.

The tool developed in this study to assess the fitness-for-purpose of analytical methods, by integrating analytical performance criteria with environmental sustainability metrics, was implemented in an Excel-based framework (Microsoft 365, Redmond, WA, USA). (Supplementary File S1), in which VBA macros were developed to automate calculations and data processing. The tool is based on three steps, as depicted in the workflow shown in Figure 1: powder blue for step 1 “Selection aim of method”, pinkish light red for the step 2, “Assessment of analytical performance”, and green for step 3, “Greenness calculation”. Each step corresponds to a group of sheets identified by the same color code.

The first step requires the user to define the method application field in sheet 2 (2. Selection aim of method) according to the classification provided in SANTE/11312/2021 v2 (Section G8) [14], choosing between (1) quantitative method (QM) and (2) screening method (SM) (see Figure 1). Specifically, a SM is intended to detect the presence or absence of an analyte in a sample, typically at or above a predefined concentration threshold with a confidence level of 95%. Based on the selection of the method application field, the user user has to click the “Go to the Analytical Performance Sheet” button to be redirected to specific analytical performance sheets (3. AP_QM or 5. AP_SM, depending on the selected method aim), thereby starting the second step of the workflow.

In the second step, instrumental parameters and validation data specific to the selected commodity have to be provided as input to complete the calculation of analytical performance parameters. The tool is designed to be applied to a single commodity group at a time, as differences in matrix composition and the presence of interfering substances can affect the efficiency of extraction and the reliability of analytical results, as specified in the SANTE/11312/2021 v2 document. Once the table is completed, the user has to click on the “Analytical Performance Analysis” button, and the tool will provide an Excel sheet with the evaluation results of analytical performance (4. Score_AP_QM or 6. Score_AP_SM).

The last step (Step 3) for the user is to complete the table in the “Greenness Calculation” Excel sheet to calculate environmental sustainability assessments. The table faithfully reproduces the AGREE interface, which operationalizes the 12 principles of green analytical chemistry [29]. For each principle, the user selects the option that best describes the method. The AGREE score is then automatically calculated for each principle. Moreover, a color-coded pictogram will be shown by clicking the “Analysis of GREEnness Performance” button.

### 2.2. Assessed Analytical Method

Three validated methods were assessed and compared for the analysis of glyphosate in high-starch or high-protein commodities with low water and fat content (Commodity Group 5 according to the SANTE guideline; Annex A [14]).

- Method 1 (LC-MS/MS—quantitative): Based on the QuPPe procedure followed by analysis of LC-MS/MS. This method allows the analysis of polar pesticides such as glyphosate and its metabolites. It is officially recognized by the EU Reference Laboratory for Pesticide Residues in Food with Single Residue Method (EURL-SRM) [13].
- Method 2 (FI-MS/MS—quantitative): Employs FI (i.e., direct injection into the mass detector, without chromatographic separation) combined with MS/MS [22].
- Method 3 (LFA—screening): Based on a commercial immunochromatographic assay (lateral flow assay) that relies on antigen–antibody recognition specific to glyphosate [21].

In addition to the manual LFA procedure (Method 3a), a fully automated version of the immunochromatographic assay procedure (Method 3b, automated LFA—screening) was assessed to evaluate the ecological impact of method automation.

### 2.3. Materials and Reagents

Pesticide standard solutions of glyphosate (1000 mg/L water/acetonitrile (9:1, *v*/*v*)), AMPA (100 mg/L water/acetonitrile (9:1, *v*/*v*)), ethephon, HEPA (ethephon metabolite), glufosinate, MPPA (glufosinate metabolite), *N*-acetyl-glufosinate (glufosinate metabolite), phosphonic acid, and *N*-acetyl glyphosate neat were purchased from Lab Instruments Srl (Castellana Grotte, Italy). Ethylenediaminetetraacetic acid disodium salt dihydrate (EDTA) ≥ 99% was obtained from Merck (Darmstadt, Germany). Methanol (MeOH) and Acetonitrile (ACN) were obtained from Carlo Erba Reagents Srl (Milan, Italy). All solvents used were of LC–MS or analytical grade. Unless otherwise specified, water purified by a Milli-Q system (Millipore, Merck KgaA, Darmstadt, Germany) was used for sample preparation and analysis.

Specifically, for the LFA analysis, a stock solution of glyphosate was prepared at a concentration of 90 µg/mL for glyphosate, and a matrix-matched calibration curve in wheat was prepared by serial dilution of a wheat extract at 0.6 µg/mL (corresponding to 6.0 mg/kg) with blank wheat in the following concentration range: 0.05, 0.09, 0.19, 0.38, 0.75, 1.50, and 3.00 mg/kg. Each level was analyzed in triplicate by an immunochromatographic test.

### 2.4. Method 1: LC-MS/MS—Quantitative

The extraction procedure was based on the QuPPe-PO-Method (v12.3) [16]. First, 5 g of wheat was extracted with 9 mL of deionized water by shaking for 30 min. Thereafter, 100 µL of isotopically labeled internal standard (ILIS) mix, methanol containing 1% formic acid (10 mL), formic acid (0.1 mL), and aqueous solution containing 1% EDTA (1 mL) were added. The mixture was centrifuged at 4000 rpm for 15 min, frozen at −80 °C for 15 min, and centrifuged at 15,000× *g* for 10 min at 4 °C. Then, 2 mL of the sample extract was diluted with 2 mL of acetonitrile. Samples were injected into the LC-MS/MS system consisting of an ExionLC system interfaced to a 6500 QTRAP mass spectrometer (Sciex, Foster City, CA, USA) equipped with an electrospray (ESI) source. The column and the MS condition were as described in QuPPe-PO-Method (v12.3 method 1.6b).

### 2.5. Method 2: FI-MS/MS—Quantitative

Samples were prepared as described in Ciasca et al. [22]. Briefly, 2.5 g of chickpeas and wheat (group 5) were extracted with 10 mL of deionized water by shaking for 30 min. The samples were then centrifuged (15 min, 2500× *g*) and filtered through a glass microfiber filter. Then, 1 mL of the filtered extract (corresponding to 0.25 g) was passed through an Oasis^®^ HLB column (activated and conditioned according to the manufacturer’s instructions) and collected into a 3 mL glass vial. The purified extract was then diluted with distilled water. Dilution factors of 1:5 were applied. The diluted extract was filtered through a 0.22 µm filter. The final test sample was prepared by adding 40 µL of ILIS (1,2-^13^C_2_ ^15^N-glyphosate) solution to 200 µL of the filtered extract. The test sample (2 µL) was injected into an MS/MS instrumental setup consisting of an Acquity UPLC system (binary pump and microautosampler, Waters, Milford, MA, USA) interfaced to an API 5000 mass spectrometer (AB Sciex, Foster City, CA, USA) equipped with an electrospray (ESI) source. The FI-MS/MS detection parameters were described in Ciasca et al. [22].

### 2.6. Method 3a: LFA—Screening

Glyphosate analysis by lateral flow assay was carried out on a prototype lateral flow assay provided by VICAM, a Waters business [24]. Samples were analyzed according to the manufacturer’s instructions.

Three grams of test sample (wheat) were weighed into a Vertu test extraction tube with 30 mL of water and blended for 2 min. The sample was filtered, and 1 mL (corresponding to 0.1 g of matrix) was transferred into a 2 mL plastic test tube with a cap. 0.1 mL of reagent A (buffer solution, provided in the kit) and 0.1 mL of reagent B (derivatization solution, provided in the kit) were added, and the sample was vortexed for 15 s. The final sample extract was left at room temperature for 5 min; subsequently, 0.1 mL were pipetted onto the lateral flow device and allowed to run for 5 min at room temperature. Afterwards, the lateral flow device was immediately placed into the reader’s holder. Intensities of the test line and control line developed on the strip membrane were measured using a reading system and specific software provided by the supplier. The ratio of the test and control lines was converted into glyphosate mass fraction through a lot-specific calibration curve, prepared in-house and uploaded into the reader via a QR code.

### 2.7. Method 3b: Automated LFA—Screening

**Figure 2:**
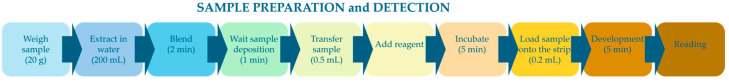
Workflow of the ALFA (automatic lateral flow analysis) NANO system (https://www.safefood.it/#alfa-nano, accessed on 22 January 2026) implemented for glyphosate analysis.

A fully automated procedure was implemented on the ALFA (automatic lateral flow analysis) NANO system (https://www.safefood.it/#alfa-nano, accessed on 22 January 2026). The platform, currently used for automated aflatoxin analysis via lateral flow tests, was slightly adapted for the analysis of glyphosate. Specifically, twenty grams of sample was weighed into a tube extraction, and the ALFA NANO system automatically performed the extraction and analysis according to the workflow shown in Figure 2. The power requirement of the instrument is 0.5 KW.

## 3. Results

### 3.1. Tool Description: Fitness-for-Purpose Evaluation Framework

This study proposes a tool for assessing the fitness-for-purpose of pesticide analytical methods, considering both their analytical performance and environmental impact. The tool was developed as an Excel-based framework comprising seven interconnected worksheets supported by VBA macros, providing a user-friendly interface that integrates method validation, compliance checking, and AGREE score calculation. The included sheets are: 1. Instructions (provides step-by-step guidance for using the tool), 2. Selection Aim of Method (allows users to define the intended purpose of the method), 3. AP_QM (analytical performance assessment of QM), 4. Score AP_QM (Result of analytical performance assessment for QM), 5. AP_SM (analytical performance assessment for SM), 6. Score AP_SM (Result of analytical performance assessment for QM), 7. Greenness Calculation. The layout of the sheets and tables follows the color-coded scheme reported in Figure 1: sheets 1–2 in powder blue, sheets 3–6 in pinkish light red, and sheet 7 in green. In the first step, users define the aim of the method (Step 1, Figure 1) by selecting the intended purpose in the “2. Selection Aim of Method” sheet, highlighted in powder blue. Based on this selection, the tool automatically directed users to the second step, which involves the assessment of analytical performance. Specifically, the 3. AP_QM sheet is shown for QM, whereas the 5. AP_SM is shown for SM (Step 2, Figure 1). Each worksheet guides the users through the input of the parameters describing the analytical method (method typology, MS detector, number of analytes), as well as the threshold value and the experimentally obtained validation parameters. The threshold value and the validation parameters differ between the two worksheets according to the intended purpose of the method: AP_QM requires MRL and 13 analytical performance parameters (Table 2a), whereas AP_SM requires the level of interest (LOI) and three analytical performance parameters (Table 2b). When multiple metabolites are included in the MRL definition, they should be identified by the user. Although the SANTE guidance does not explicitly require the limit of detection (LOD), it was included in the analytical performance parameter for quantitative methods because of its relevance for risk assessment. For example, EFSA’s 2020 report considers scenarios in which non-quantifiable residues (<LOQ) are assigned ½ LOQ or LOQ for exposure modeling. This highlights the importance of detecting low-level contamination. Such a conservative approach emphasizes that even non-quantifiable residues can contribute to consumer exposure, making the inclusion of LOD a valuable parameter for more comprehensive risk modeling (middle and upper bound approaches).

**Figure 3:**
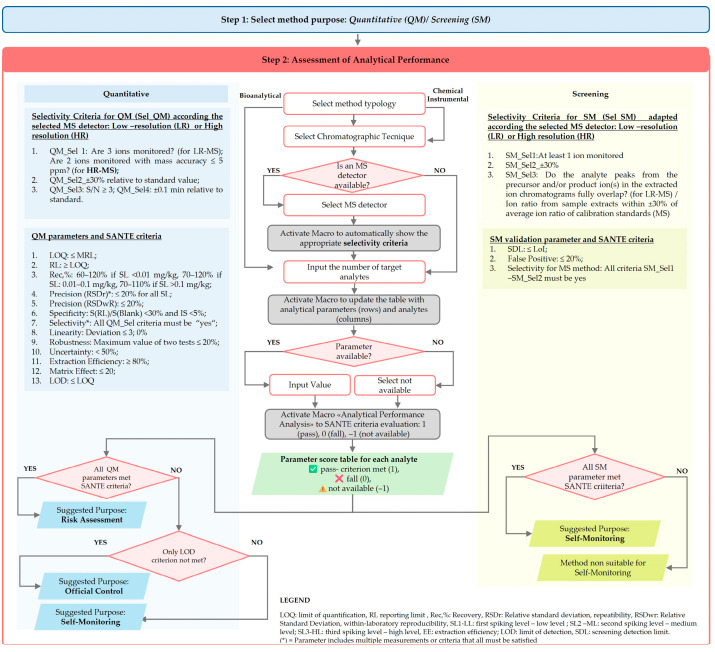
Flowchart of the tool used to evaluate the fitness-for-purpose of analytical methods. The first two steps of the process are shown. In Step 1, the words “Quantitative Method (QM)” and “Screening Method (SM)” are italicized to indicate selectable options. Step 2 presents the flowchart using coordinated color and shape coding: grey rectangles indicate macro-activation steps, rounded rectangles represent workflow actions, red diamonds denote decision points with Yes/No outcomes, and parallelepiped shapes indicate tool outputs. The central section displays steps common to both QM and SM. Light-blue shading (left side) highlights criteria, parameters, and workflow steps specific to QM, while yellow shading (right side) highlights those specific to SM.

The selectivity criteria required by the tool vary automatically according to both the intended purpose of the method (quantitative or screening) and the mass spectrometric approach, with distinct requirements for unit-mass and high-resolution MS instruments that reflect their different identification capabilities. For instance, for qualitative MS-based screening methods, only the minimum number of ions is required. Regarding the screening method, SDL is the only parameter used as a threshold value. According to the SANTE document, “for a screening method, the detection confidence at a given concentration level should be established based on the Reporting Limit (RL) from the validation of a quantitative method or on the Screening Detection Limit (SDL) from the validation of a qualitative method”. Consequently, the screening threshold may be expressed as RL only when a confirmatory quantitative method is available to verify both qualitatively and quantitatively the results; in the absence of such a method, SANTE requires the use of SDL. In addition to SDL, the tool also estimates the false-positive rate according to [35], even though this parameter is not explicitly mandated by the SANTE guideline [14]. Section G10 specifies that the potential for false detections should be evaluated using blank (non-spiked) samples, and it does not impose a strict numerical criterion for false positives as long as detected analytes are subsequently identified and quantified by a confirmatory method [14]. The inclusion of this metric in the tool provides valuable insight into method selectivity and contributes to both fitness-for-purpose evaluation and ecological impact assessment. The tool also allows users to select a “Not Available” option for parameters that were not calculated or are unknown, ensuring that the validation tables can be completed even when some experimental data are missing. The tool then automatically verifies compliance with SANTE requirements by dedicated VBA macros and the score is displayed directly in the respective worksheet (Score AP_QM or Score AP_SM). For both AP_QM and AP_SM, each parameter is scored as 1 if the acceptance criterion is met and 0 if the criterion fails. Results are presented in tabular form using conditional color coding to facilitate interpretation: a green check mark (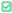) indicates that the criterion is met, a red cross (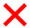) signals a failed criterion, and a warning symbol (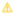) highlights parameters that are missing or not evaluable. The same symbols were used in correspondence to the row labeled “Analyte complying with all criteria” to summarize the overall performance of each analyte. If all parameters meet the SANTE criteria, the tool suggests the quantitative method as suitable for risk assessment. If all parameters are met except for the LOD, the quantitative method is considered suitable for official control. Otherwise, the method is recommended for self-monitoring only. Figure 3 presents a detailed flowchart of the first two steps illustrating all decision points and the threshold values applied to each parameter (based on SANTE criteria).

In the final step, assessment of environmental performance (Figure 1, Step 3), the tool requires the completion of Table 3, which reports the twelve principles used for the calculation of the AGREE score.

Depending on the principle, the tool prompts the user to input specific data, such as for Principles 2, 9, and 10, where quantitative information is required, or to select an appropriate option from a predefined list for the remaining principles. In the case of Principle 1, for instance, the tool requires users to select the most appropriate analytical approach among the following options: remote sensing without sample damage; remote sensing with little physical damage; non-invasive analysis; in-field sampling and direct analysis; in-field sampling and on-line analysis; on-line analysis; at-line analysis; external sample pre-treatment and batch analysis (reduced steps); or external sample pre-treatment and batch analysis (large steps). The final output is a table that shows, for each principle, a score ranging from 0 to 1. Each score is also represented with a color-coded background, following a traffic-light scheme that makes it easy to interpret at a glance. Red indicates low performance (score = 0), orange to yellow represents moderate performance (score 0–0.5), and yellow to green indicates high performance (score 0.5–1) In addition, clicking on “AGREE score” displays a pictogram that visually summarizes the overall results. This approach allows users to quickly understand the environmental performance of each principle and the overall assessment.

### 3.2. Case Study: Methods’ Assessment

**Figure 4:**
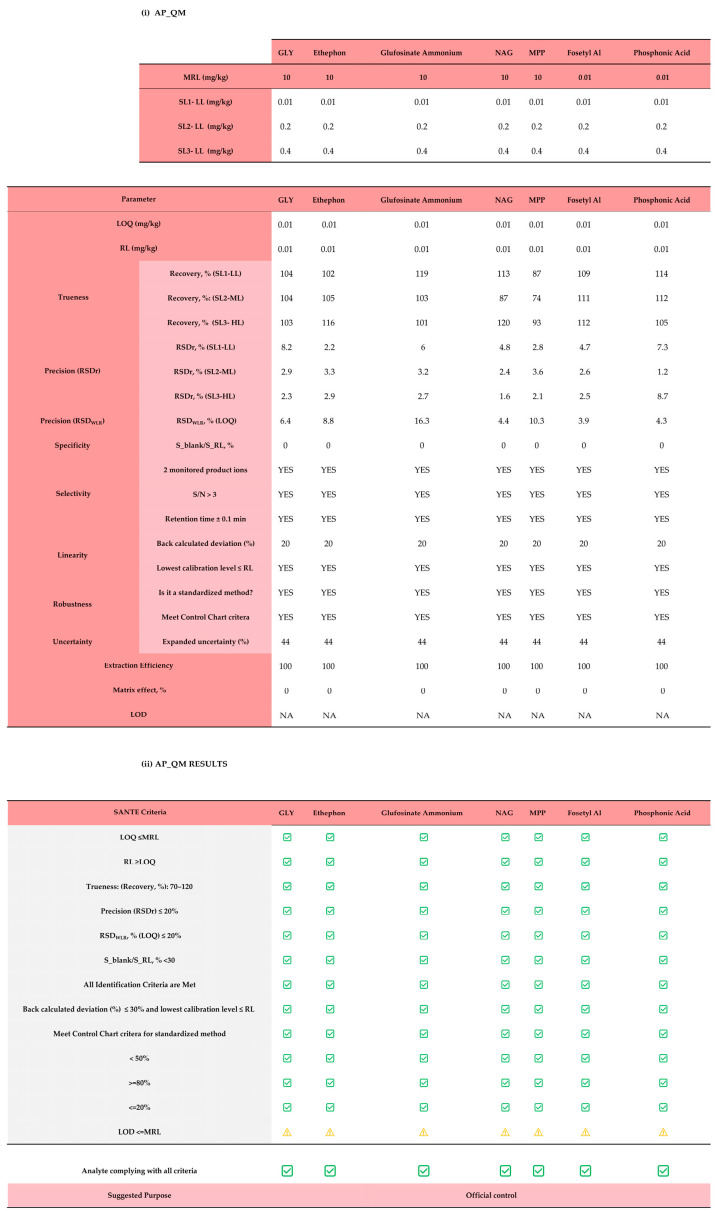
Assessment of the analytical performance (AP) of method 1 (LC-MS/MS—quantitative): (i) input data in “3. AP_QM” sheet; (ii) output data in “5. Score AP_QM” sheet. NA = data not available; (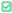) = criterion is met; (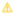) = missing parameter. Red shading highlights Step 2 (Assessment of Analytical Performance), grey shadow in (ii) AP_QM result denotes the SANTE acceptance criteria that each parameter must meet.

The case study consisted of testing the tool’s applicability to assess three analytical methods, then comparing the results. The first assessed method (Method 1—LC-MS/MS quantitative) is based on the QuPPE procedure (version 12.3) followed by LC-MS/MS detection [16]. This method is aimed at quantitative analysis (Step 1). Validation data were provided by an official control body applying the procedure described for official (routine) controls. The input data entered in the tool, as well as the results of the analytical performance evaluation (Step 2), are shown in Figure 4.

The LC-MS/MS method demonstrated analytical performance fully complying with the SANTE criteria for quantitative methods for all tested analytes. The limits of quantification (LOQs) were consistently below the MRLs, ensuring adequate sensitivity. Recovery rates for all analytes fell within the acceptable range of 70–120%, indicating high trueness, while precision metrics (RSDr and RSDwR) remained well below the 20% threshold, confirming the method’s reliability and reproducibility. No matrix interference was detected, and unambiguous identification was achievable by monitoring multiple product ions and maintaining signal-to-noise ratios above 3. This indicates adequate specificity and selectivity. The method showed a suitable linearity range with back-calculated concentration deviations under ±20%. In addition, being a standardized method, it does not require an assessment of robustness but only performance verification through standardized procedures and control charts (Jean control chart and Howarth control chart). Uncertainty values were below 50%, further supporting the reliability of the results. Extraction efficiency was 100%, and matrix effects were negligible thanks to the use of ILIS, which compensates for any potential losses during sample preparation and matrix interference on the signal. The limits of detection (LOD) were not calculated. Based on these results, the tool suggests that this method is suitable for official control and regulatory compliance.

**Figure 5:**
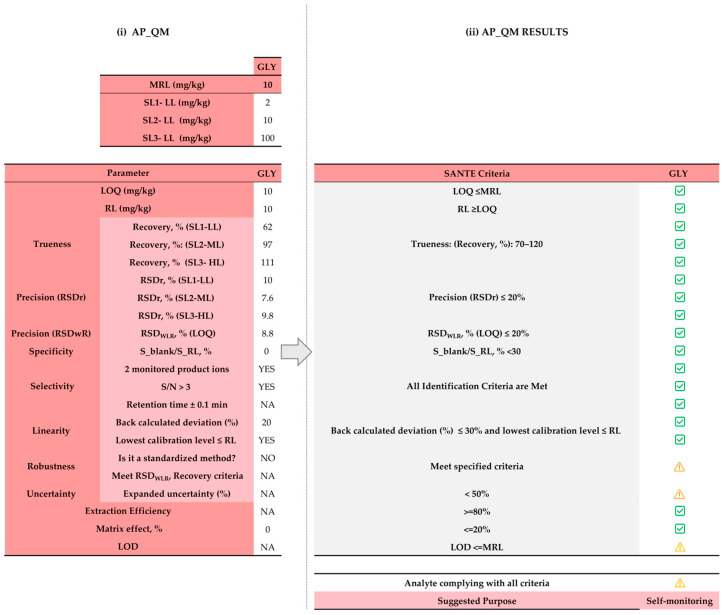
Assessment of analytical performance of method 2 (FI-MS/MS—quantitative): (i) input data in “3. AP_QM” sheet; (ii) output data in “4. Score_AP_QM” sheet. NA = data not available; 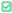 = acceptance criterion met; (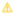) = parameters missing or not evaluable. Red shading highlights Step 2 (Assessment of Analytical Performance), grey shadow in (ii) AP_QM result denotes the SANTE acceptance criteria that each parameter must meet.

The second method was also intended for quantitative analysis, and validation data were generated by the authors in a previous study [22]. However, the method is not standardized. Consequently, during the validation phase, it would be necessary to evaluate its robustness—yet this parameter, as well as the associated uncertainty, is not reported. Trueness was assessed by comparing the results obtained with this method with those obtained using an official, accredited method performed by an official control laboratory [22]. Although the method does not include chromatographic separation, potential matrix effects and isobaric interferences were assessed through interference challenge experiments, confirming that the method can reliably distinguish glyphosate from co-extracted matrix components and other compounds. Finally, the lack of uncertainty values and data on robustness affects the overall fitness-for-purpose; therefore, the method is recommended primarily for self-monitoring (Figure 5).

**Figure 6:**
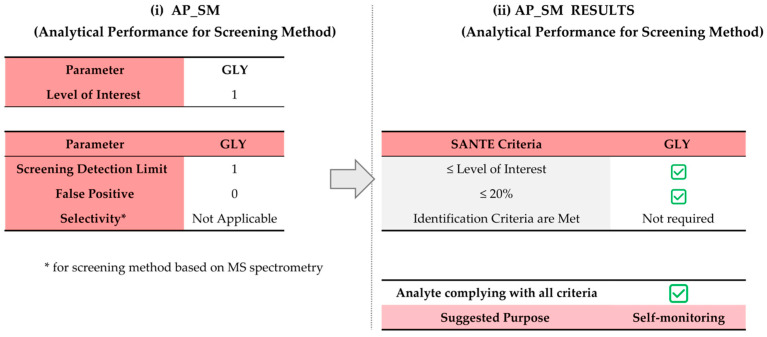
Assessment of analytical performance (AP) of method 3 (LFA—qualitative): (**i**) input data in “5. AP_SM sheet”; (**ii**) output data in “6. Score_AP_SM” sheet. 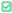 = acceptance criterion met; Red shading highlights Step 2 (Assessment of Analytical Performance), grey shadow in (ii) AP_SM result denotes the acceptance criteria that each parameter must meet.

The last method was a qualitative screening method. Screening methods do not provide reliable quantification; instead, they indicate whether a sample exceeds a predefined threshold. According to the SANTE guideline [14], any suspect positive screening result must be analyzed using a more selective and accurate confirmatory method before the result can be used for official control purposes. Therefore, a qualitative screening method alone cannot be used for official control, as it does not provide unquestionable identification and quantification. It must always be complemented by a confirmatory method to validate any suspect positive findings and ensure compliance with regulatory limits. For the LFD qualitative method, a level of interest (LOI) of 1 mg/kg was selected, since performance must be assessed at concentrations equal to or lower than the MRL of 10 mg/kg for wheat. For this method, the SDL was determined by analyzing 20 blank matrix samples spiked at 1 mg/kg. In parallel, 20 non-spiked blank samples were analyzed to assess the background signal and potential false detections. All spiked samples were consistently detected, while no detections were observed in the blank samples. Therefore, the SDL was established at 1 mg/kg. Based on this SDL and the qualitative nature of the procedure, the decision-support tool classified the method as suitable for self-monitoring, in accordance with its intended use as a screening method (Figure 6). However, if confirmatory analyses were carried out on positive samples, the method can also be applied within the framework of official control. 

**Figure 7:**
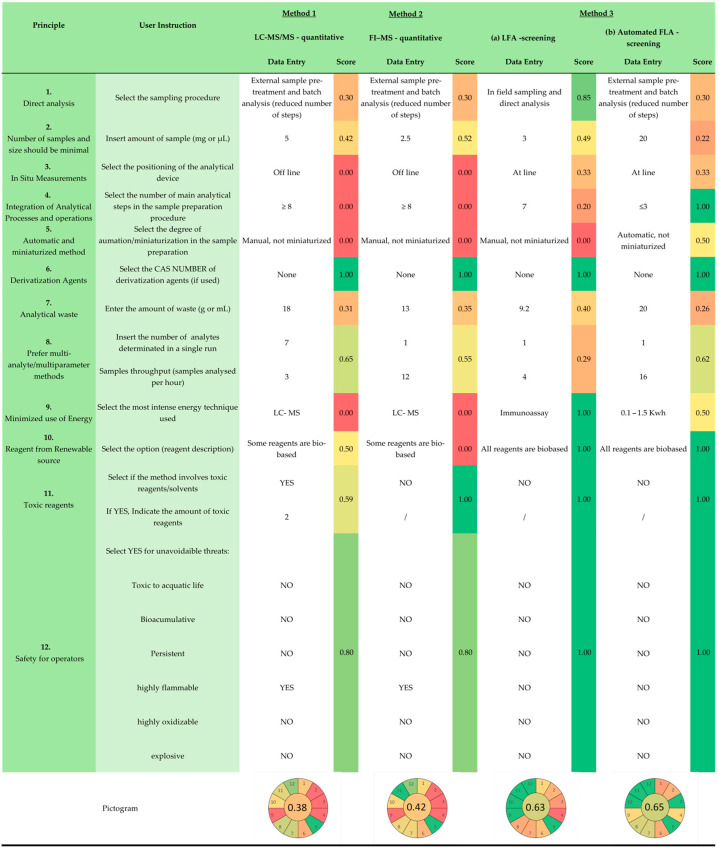
Result for Step 3—greenness calculation of method 1 (LC-MS/MS—quantitative), method 2 (FI-MS—quantitative), method 3a (LFA—screening), and method 3b (automated LFA—screening). Each score is shown with a color-coded background using a traffic-light scheme: red indicates low performance (score = 0), orange to yellow indicates moderate performance (score 0–0.5), and yellow to green indicates high performance (score 0.5–1). Green shading highlights Step 3 (Greeness Calculation).

The last step of the assessment, focused on greenness evaluation, revealed clear differences in the operational and environmental performance of the three selected methods, as shown in the summary table in Figure 7. 

Direct analysis (Principle 1) favored field LFA (0.85), while the other methods (LC-MS/MS, FI-MS, and automated LFA) scored 0.30. For Principle 2, an ideal sample should weigh less than 0.1 g, and none of the methods used meet this requirement (score from 0.22 to 0.52). Lower scores were observed for principle 3: in situ measurements (0.33 for LFAs, both manual and screening, and 0.00 for the other methods). Integration of analytical processes (Principle 4) was highest for automated LFA (1.00, ≤3 steps), and automation and miniaturization (Principle 5) favored automated LFA (1 and 0.50, respectively). Derivatization solvents were avoided (Principle 6) in all of the assessed methods; therefore, the maximum score was achieved (1.00). The highest score for principle 8 (multi-analyte/parameter detection) was observed in LC-MS/MS (0.69) and automated LFA (0.62) despite different operational characteristics. Principle 8 reflects a method’s multi-analyte capability, calculated as the total number of analytes analyzed per hour (analytes per run × samples per hour). LC-MS/MS analyzed seven analytes simultaneously but had a lower sample throughput, while automated LFA analyzed one analyte at a time but processed sixteen samples per hour.

Energy efficiency (Principle 9) favored only LFA (1.00). Sustainable reagent use (Principle 10) scored 1.00 for LFAs (water as a renewable solvent) and 0.50 for LC-MS/MS and FI-MS. Toxicity (Principle 11) was slightly lower for LC-MS/MS (0.59) due to acetonitrile, whereas FI-MS and LFAs scored 1.00. Operator safety (Principle 12) followed a similar trend: LFAs (1.00) vs. LC-MS/MS and FI-MS (0.80) due to methanol use. Overall, automated LFA combined the best environmental and operational performance, while LC-MS/MS and FI-MS offered high analytical flexibility but with higher energy demand, toxic reagents, and complex workflows.

## 4. Discussion

The tool developed in this study was designed to support operators in verifying and improving method performance, merging analytical performance criteria, and improving environmental performance. The alignment with the intended purpose of each method is crucial, as analytical performance parameters acquire practical meaning only when evaluated in relation to the method’s scope. For this reason, the tool first guides the user in classifying the method either as quantitative, which provides a numerical result, or as screening, which determines whether an analyte is present above or below a predefined threshold. This initial classification determines the performance criteria to be checked in accordance with SANTE/11312/2021 v2 [14].

The tool is designed to evaluate and compare fitness-for-purpose methods in one commodity group at a time, in accordance with SANTE/11312/2021 v2. Since matrix composition and the presence of interfering substances strongly influence extraction efficiency, sensitivity, and analytical reliability, direct comparison of method performance across different commodity groups is not recommended. A commodity group selection option has been implemented in the tool, ensuring that analytical performance is referred to and compared in relation to the selected commodity group. As a case study, in this work, a high-starch or high-protein commodity with low water and fat content (Commodity Group 5 according to the SANTE guideline, Annex A [14]) was selected.

As demonstrated by the comparative assessment in this case study, the quantitative methods intended for official control require a substantial validation effort. To comply with the stringent requirements of regulatory monitoring and risk assessment, the operator must generate extensive datasets covering trueness, precision, selectivity, uncertainty, robustness, etc. LC-MS/MS methods, due to their intrinsic sensitivity and selectivity, allow for fulfilling these requirements and therefore remain the reference analytical techniques for confirmation and enforcement. Within the group of quantitative methods, the FI-MS/MS approach has emerged as a promising option. Although the assessed method is currently more suitable for self-monitoring applications due to the lack of data on robustness and measurement uncertainty, the method has the potential to meet the requirements of official control, provided that these performance characteristics are appropriately established and verified. It should be noted that the fitness-for-purpose of analytical methods must be assessed with respect to the applicable residue definition. In the case of glyphosate, there is currently no direct regulatory requirement to analyze AMPA or other metabolites for enforcement in cereals or for risk assessment [10]. In the FI-MS/MS method, only glyphosate was validated, whereas the LC-MS/MS method validation data also included its relevant polar metabolites, including AMPA, in order to address EFSA’s request [36] to monitor metabolites for dietary risk assessment, thereby covering the compounds that may be included in future MRL legislation and risk assessments.

Screening methods, as exemplified by the LFA, require less stringent validation criteria, as their objective is to detect whether an analyte is present above or below a given threshold. Qualitative screening methods can be used in official controls, provided that positive samples are subsequently analyzed using confirmatory methods. Therefore, the evaluation of the false-positive rate for a screening method, although not explicitly required by the SANTE document [14], is a relevant piece of information, since a high false-positive rate increases the number of required confirmatory analyses, affecting both the method’s fitness-for-purpose and its overall environmental footprint. Negative samples, on the other hand, do not require confirmation.

Comparison of greenness indicators highlighted substantial differences among the three analytical approaches. LC-MS/MS and FI-MS/MS showed lower greenness performance (0.38 and 0.42, respectively) compared to LFA (0.63 for LFA and 0.68 for automatic LFA).

The lower sustainability of LC-MS/MS and FI-MS/MS is primarily attributed to offline sample preparation, the generation of larger volumes of chemical waste, and the significant energy consumption associated with MS instrumentation, in addition to the use of solvents not derived from renewable sources. Offline sample preparation is highly matrix-dependent, directly affecting analytical performance and environmental impact. High-starch commodities, such as wheat, require more complex preparation—including homogenization, extraction, and cleanup—to minimize matrix interferences and ensure accurate quantification. In contrast, high-moisture matrices like fruits and vegetables allow simpler extraction with minimal cleanup, resulting in lower matrix effects and higher reproducibility. Thus, evaluating method suitability and sustainability requires focusing on a single representative commodity group, as applying the same procedure to matrices belonging to different commodity groups can yield markedly different results. It should be noted that the MS method for glyphosate analysis (LC-MS/MS and FI-MS/MS) requires solvents for both extraction and the analytical process. In contrast, the LFA approach uses water (a renewable solvent) for the extraction, and no solvent is required for the analysis. In this respect, it is worth mentioning that most of the commercially available LFAs for food contaminants employ water-based extraction, avoiding completely organic solvents. Employing renewable methanol or replacing it with ethanol in LC-MS/MS could potentially improve the greenness score.

It is important to note that, although the ability to analyze multiple analytes (seven analytes) represents a clear advantage of the LC-MS/MS method, it only partially offsets its lower environmental score compared to the other evaluated methods.

The LFA method (Method 3a) emerged as a green technique due to its intrinsically simple workflow. LFAs enable direct, in-field measurements, require minimal sample handling, operate without toxic reagents, and consume very little energy. Additionally, they avoid the use of derivatization agents (Principle 6) and enhance operator safety (Principle 12) owing to the absence of toxic and highly flammable reagents.

To illustrate how workflow modifications may influence sustainability, an automated version of the lateral flow method (Method 3b) was evaluated to highlight the specific impact of automation on greenness. Automation improved process integration, reduced operator intervention, and allowed higher sample throughput, although it required greater energy consumption.

Finally, none of the methods achieves the ideal sample size (<0.1 mg) to minimize environmental impact. The use of micro-samples in food residue monitoring is precluded by the need to balance analytical sensitivity with sample representativity. Although Directive 2002/63/EC [37] and SANTE/11312/2021 v2 [14] guidance do not specify a fixed mass for the analytical test portion, they state that the test portion must be representative of the homogenized laboratory sample, ensuring that the analytical results accurately reflect the distribution of pesticide residues throughout the entire sample.

Overall, the comparison highlights a clear trend: methods based on simple, low-energy, and low-waste analytical formats naturally align with green analytical chemistry principles. High-performance mass-spectrometry-based approaches are indispensable for quantitative and confirmatory purposes, but their environmental footprint is inherently larger. These results emphasize the value of targeted workflow optimization, reducing preparation steps, minimizing waste, and improving energy efficiency as a strategy to enhance the sustainability of even the most advanced analytical techniques.

The proposed decision-support tool provides an objective and structured evaluation of analytical methods by integrating fitness-for-purpose, in accordance with SANTE guidelines, with environmental performance assessed through the AGREE metric. Its assessment reflects the current state of analytical workflows and commercially available materials. For mass spectrometry-based methods, analytical-grade solvents are still predominantly fossil-based due to strict purity and stability requirements. Although renewable solvents—such as methanol from CO_2_ or biomass—offer a promising route to reduce environmental impact, their routine use is currently limited. Solvent renewability is already captured under Principle 10 of AGREE, so future adoption of renewable solvents would automatically improve scores without requiring structural changes to the tool.

Similarly, the environmental evaluation of screening methods such as LFAs reflects the current design of commercial devices, typically using single-use plastic components. Future innovations in recyclable, biodegradable, or plastic-free LFA formats could further enhance sustainability, including waste reduction (Principle 7), and would be directly reflected in higher AGREE scores.

The tool is intentionally flexible and forward-looking, allowing future improvements without altering the scoring logic. This adaptability ensures the tool remains applicable as analytical chemistry evolves toward more sustainable and innovative solutions.

## 5. Conclusions

The proposed decision-support tool provides laboratory operators with a structured, user-friendly, and objective methodology for evaluating analytical approaches, integrating analytical performance with compliance with EU SANTE regulations and environmental impact. Its application can support method selection by balancing reliability and sustainability. Future use is expected in the analysis of mycotoxins, antibiotics, and allergens, where mass spectrometry and rapid screening methods are commonly employed for routine testing and official control. Overall, this approach fosters efficient, reliable, and environmentally responsible monitoring in the food and feed sectors.

## Figures and Tables

**Table 1:** Overview of the main method for glyphosate detection in food of plant origin.

Target Analyte	Food Matrix	Extraction	Derivatization/Clean up	Analytical Method	Limit of Quantification (mg/kg)	Reference
GLY, PP *	Plant Origin Foods	QuPPe-PO	No	LC-MS/MS (IS)	0.1 (lentils) 0.02 (barley; apple)	[16]
GLY, AMPA, GLUF	Apple, mushrooms, lentils, wheat	QuPPe-PO	No	IC-MS/MS (IS)	0.01	[17]
GLY, GLUF	Milk-based baby food, PO Food	Acidified H2O/MeOH and DCM.	Derivatization with FMOC; SPE	LC-MS/MS (IS)	0.005	[18]
GLY, AMPA	Cereals, vegetables, fruits	QuPPe-PO; Acidified MeOH/H2O + EDTA	No derivatization, d-SPE	IC-MS/MS (IS)	Not specified	[19]
GLY	Green coffee beans	DCM and H2O.	No	LC-MS/MS (IS)	0.5	[20]
GLY	Rice (brown, white)	H2O/MeOH (70/30)	No	ICP-MS/MS (IS)	0.009 (white); 0.046 (brown)	[21]
GLY	Chickpeas	H2O	SPE	FI-MS/MS (IS)	2	[22]
GLY	Soybeans, corn	H2O	No	Lateral flow immunoassay	visual detection limit: 1 (soy), 0.2 (corn) Cut-off: 50 (soy); 5 (corn)	[23]

GLY: glyphosate, PP: polar pesticides * including ethephon, 2-hydroxyethane-phosphonic acid Aminomethylphosphonic (AMPA) acid, glufosinate (GLUF), 3-(Methylphosphinico)propionic acid, *N*-acetyl-glufosinate, phosphonic acid; DCM: dichloromethane; MeOH: methanol, FMOC: 9-fluorenylmethyloxycarbonyl; EDTA: Ethylenediaminetetraacetic acid; IC-MS/MS: ion chromatography coupled with mass spectrometry; LC-MS/MS: liquid chromatography coupled with mass spectrometry; ICP-MS/MS: Inductively coupled plasma mass spectrometry; FI-MS/MS: flow-injection MS/MS; IS: Internal Standard.

**Table 2:** (**a**) Parameters and their relative definition required for the validation of quantitative methods. (**b**) Parameters and their relative definition required for the validation of screening methods.

(**a**)	
**Parameter**	**Definition**
Limit of Quantification (LOQ)	The lowest validated spiking level meeting the identification and method performance criteria for recovery and precision.
Reporting Limit (RL)	The lowest level at which residue will be reported as absolute numbers to clients or regulatory agencies.
Trueness	The closeness of agreement between the average value obtained from a series of test results (i.e., the mean recovery) and an accepted reference or true value [32]
Precision (RSDr)	Relative Standard Deviation—Repeatability of replicate measurements under the same conditions (at least five replicates).
Precision (RSDwR)	Within-laboratory reproducibility, derived from ongoing method validation/verification.
Specificity	Response of the detected signal due to the analyte, not other compounds (interferences).
Selectivity	The ability of an analytical method to distinguish the analyte of interest from other components (interferences) in the sample matrix, either by separation or by relative response of the detection system.
Linearity	Deviation of back-calculated concentration from true concentration.
Robustness	The degree of reproducibility of test results under variable conditions within the same laboratory.
Uncertainty	Parameter associated with the result of a measurement that characterizes the dispersion of values that could reasonably be attributed to the measurand [33,34].
Extraction Efficiency	Percentage of analyte recovered after the extraction process (no absolute requirement)
Matrix Effect	Influence of co-extracted matrix components on the ionization of the analyte in the mass spectrometer.
Limit of detection (LOD)	Concentration that gives an instrumental signal significantly different from the blank or background signal.
(**b**)	
**Parameter**	**Definition**
Screening Detection Limit (SDL)	The lowest concentration level at which an analyte has been detected (not necessarily meeting the MS-identification criteria) in at least 95% of the samples (i.e., an acceptable false-negative rate of 5%).
False Positive	A result wrongly indicating that the analyte concentration exceeds a specified value.
Selectivity	The ability of an analytical method to distinguish the analyte of interest from other components (interferences) in the sample matrix, either by separation or by relative response of the detection system.

**Table 3:** Principles used for the calculation of the AGREE score.

Principles
1. Apply a direct analytical technique to avoid sample treatment.
2. Use a minimal sample size and a minimal number of samples (microanalysis).
3. Perform in situ measurements.
4. Integrate analytical processes and operations to save energy and reduce the use of reagents.
5. Enable a level of automation and miniaturization.
6. Avoid the use of derivatization agents.
7. Avoid the generation of a large volume of analytical waste and ensure proper management of analytical waste.
8. Prefer multi-analyte/multiparameter methods.
9. Minimize energy consumption.
10. Use reagents from renewable sources.
11. Eliminate or replace toxic reagents.
12. Increase operator safety.

## Data Availability

The original contributions presented in this study are included in the article/Supplementary Materials. Further inquiries can be directed to the corresponding authors.

## Supplementary Materials

The following supporting information can be downloaded at https://www.mdpi.com/article/10.3390/foods15030576/s1, Supplementary File S1: ToolKit Fit4Purpose&GreenEvaluation.
